# The Anatomy of the Tibial Nutrient Artery Canal—An Investigation of 106 Patients Using Multi-Detector Computed Tomography

**DOI:** 10.3390/jcm9041135

**Published:** 2020-04-15

**Authors:** Haidara Almansour, Eleftherios Armoutsis, Marie K. Reumann, Konstantin Nikolaou, Fabian Springer

**Affiliations:** 1 Department of Diagnostic and Interventional Radiology, Tuebingen University Hospital, Eberhard Karls University Tuebingen, Hoppe-Seyler-Str. 3, 72076 Tuebingen, Germany; haidara.al-mansour@med.uni-tuebingen.de (H.A.); Eleftherios.armoutsis@student.uni-tuebingen.de (E.A.); konstantin.nikolaou@med.uni-tuebingen.de (K.N.); 2 Department of Trauma and Reconstructive Surgery, BG Unfallklinik Tuebingen, Eberhard Karls University Tuebingen, Schnarrenbergstr. 95, 72076 Tuebingen, Germany; mreumann@bgu-tuebingen.de; 3 Department of Diagnostic Radiology, BG Unfallklinik Tuebingen, Eberhard Karls University Tuebingen, Schnarrenbergstr. 95, 72076 Tuebingen, Germany

**Keywords:** nutrient foramen, tibia, computed tomography, foraminal index, tibial fracture, stress fracture, external fixation, safe corridors

## Abstract

Radiologic evaluation of the tibial nutrient artery is clinically important as disruption of tibial blood supply is a risk factor for delayed or non-union of tibial fractures. Damage to the tibial nutrient artery canal (TNAC) may occur by a traversing fracture or iatrogenic cause in the context of pin/screw placement. Furthermore, TNAC could be misdiagnosed as a stress fracture. The aim of this study was to characterize the normal anatomy of TNAC and to delineate its gender and side-specific differences. Patients who underwent contrast-enhanced computed-tomography encompassing the pelvis and lower extremities were included. TNAC was identified with an external and internal foramen and a traversing intercortical canal. Various anatomical morphometrics were evaluated: total number of nutrient canals, angular position of the outer and inner nutrient foramina, absolute and relative position of the nutrient foramina, as well as the intercortical canal length with respect to tibial length. The majority of patients of both genders had only one tibial nutrient canal, multiple canals or complete absence were rare. In most cases, the outer nutrient foramen was found on the posterolateral aspect of the upper-third of tibia at about 32% of tibial length; the inner foramen was found at the middle third of the tibia (41% of tibial length). The course of nutrient canal was mostly cranio-caudal with a small, but significant difference in relative canal length: 8.5% vs. 10% of tibial length for females and males, respectively. The angular location of the outer and inner foramen was between 20–30° and did not reveal a statistically significant difference between genders. No statistically significant side specific differences were found for all analyzed parameters and both genders. The clinical relevance of this anatomical study pertains to establishing “safe corridors” of pin/screw insertion in the context of surgical management of tibial fractures in order to avoid iatrogenic disruption of tibial blood supply.

## 1. Introduction

The vascular supply of the tibia comprises three interrelated nutritional systems which are vital to osseous integrity not only in embryologic and fetal development, but also during early phases of ossification and later on in adults [1,2,3]. In addition to the diaphyseal tibial nutrient artery (TNA), there are also small perforating arteries entering the bone at the metaphyseal level and less frequently at the epiphyseal level of the tibia resulting in an at least redundant blood supply of metaphyses and epiphyses. The periosteum also provides blood supply but can only support the outer-third of the cortical bone while the TNA supplies the inner two-thirds of the cortex and traverses the cortex for almost 5 cm [4,5]. Furthermore, the TNA provides more than 70% of intraosseous blood supply during childhood [3]. Hence, medullary ischemia and consequently, growth impairment due to a less perfused epiphyseal plate, could ensue in case of disruption of this dominant artery [3].

A detailed characterization of the TNA and its course through the diaphyseal cortex using Computed Tomography (CT) has several surgical and radiological implications. In the setting of traumatic tibial fractures, disruption of the TNA by the fracture itself has been described as a risk factor for delayed or non-union [5]. Iatrogenic disruption by internal or external fixation devices may have a similar effect [4]. Therefore, anatomical studies have already been performed investigating the effect of medullary nailing and internal fixation devices, e.g. plates and screws, on the TNA integrity [6]. In addition, the tibial nutrient artery foramen (TNAF) or tibial nutrient artery canal (TNAC) may serve as a weak point at which longitudinal fractures could nucleate, especially if it was atypically located at the anterior aspect of the tibia [7]. Furthermore, TNAF and TNAC could also be misdiagnosed as a stress fractures by the unwary radiologist [8].

Previous anatomical studies embarked on characterizing the foramina of TNAC. However, the methods utilized in most of these studies can be considered much less sensitive than CT such as the use of naked eye, magnifying lenses, Vernier calipers, osteometric boards, tapes or digital radiography [1,3,9,10,11,12,13]. Furthermore, cadaver bones are susceptible to abrading which could affect anatomical features and researchers were often unable to delineate the gender or age of the persons whose tibiae were analyzed [14]. To the best of our knowledge, only one study utilized CT but they did neither delineate the angular position of the canal nor did they evaluate gender or side differences which are clinically relevant factors in the setting of preoperative planning for placement of internal/external fixation devices [14].

Thus, the aim of this study was to provide a detailed topographic analysis of the TNAC as well as its inner and outer foramen in vivo, and to evaluate gender and side specific differences in detail.

## 2. Materials and Methods

### 2.1. Study Population

**Figure 1:**
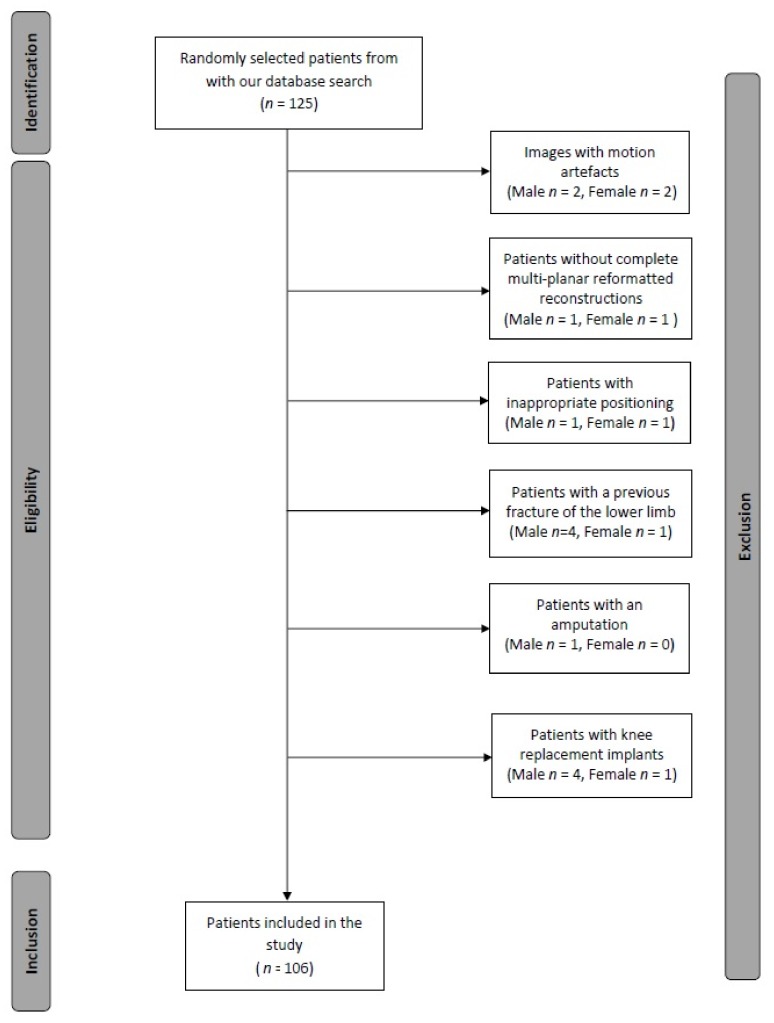
Flow diagram of the inclusion/exclusion process.

This retrospective study was approved by the institutional review board. The requirement for informed consent was waived. Patients who underwent contrast-enhanced computed tomographic angiography (CTA) encompassing the pelvis and lower extremities were included. CTA data sets of the lower limbs were used since almost all data sets display both lower legs allowing the assessment of the tibial nutrient artery and comparison of right and left sides. Furthermore, these patients most likely have a normal osseous anatomy without fractures impairing the evaluation of the tibial nutrient artery canal. Exclusion criteria were incomplete examination, inadequate image quality, or previous fracture of the lower limb. Figure 1 illustrates the inclusion/exclusion process of our patient population.

### 2.2. Imaging Protocol

All patients were scanned on a high-end Dual-Source CT-scanner (Definition Force, Siemens Healthineers, Erlangen, Germany). Acquisition parameters were as follows: dual-energy acquisition with 80 kV/Sn150 kV, reference mAs 118/59, pitch 0.7. Images were reconstructed on a 512 × 512 matrix with 3 mm slice thickness using iterative reconstruction (ADMIRE 2, Siemens Healthineers, Erlangen, Germany).

### 2.3. Anthropometric Analysis

**Figure 2:**
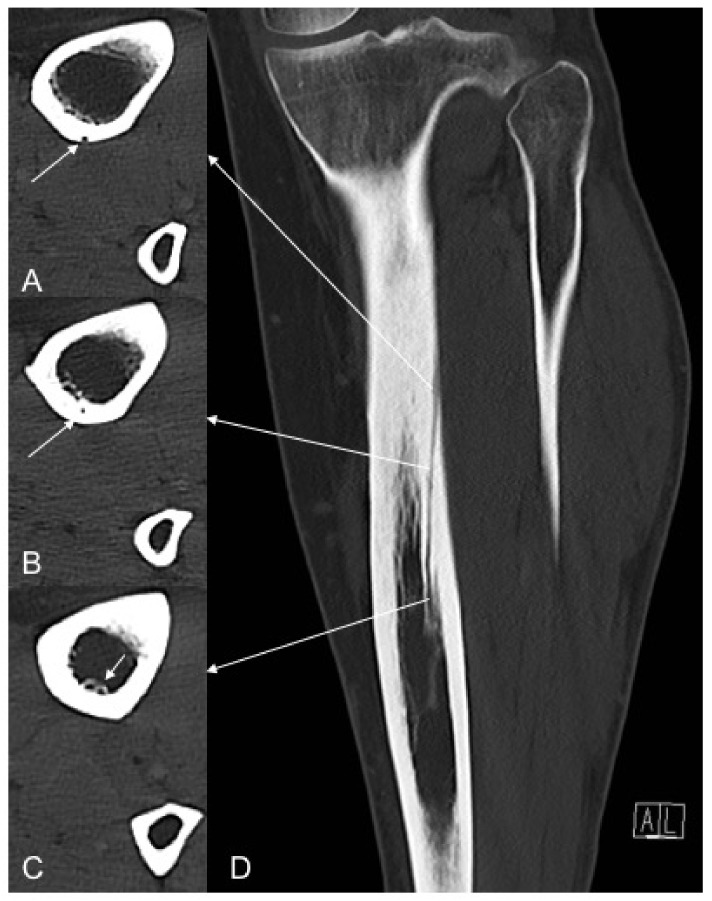
Subsequent axial (**A**–**C**) and coronal (**D**) reformatted images in bone window indicating the foramen of the nutrient artery canal on the outer cortex (**A**) (white arrows), cortical canal course (**B**) and inner cortex (**C**) (white arrows).

TNAC was identified with an outer and inner foramen and a traversing cortical canal (Figure 2).

The following morphometrics were extracted from the arterial phase reconstructions of the CTA:

-Total number and position of inner and outer nutrient foramina in relation to tibial plateau and fibula.

-Total tibial length: the distance between the medial tibial plateau and the tip of the medial malleolus.

-Foraminal index *(FI):* distance of the outer/inner foramen from medial tibial plateau relative to total tibial length. Position of the outer nutrient foramen was categorized into three groups [9,14]:

- FI (0–33.3%): Nutrient foramen is located in upper third of the tibia.
- FI (33.3–66.7%): Nutrient foramen is located in middle third of the tibia.
- FI (66.7–100%): Nutrient foramen is located in lower third of the tibia.

**Figure 3:**
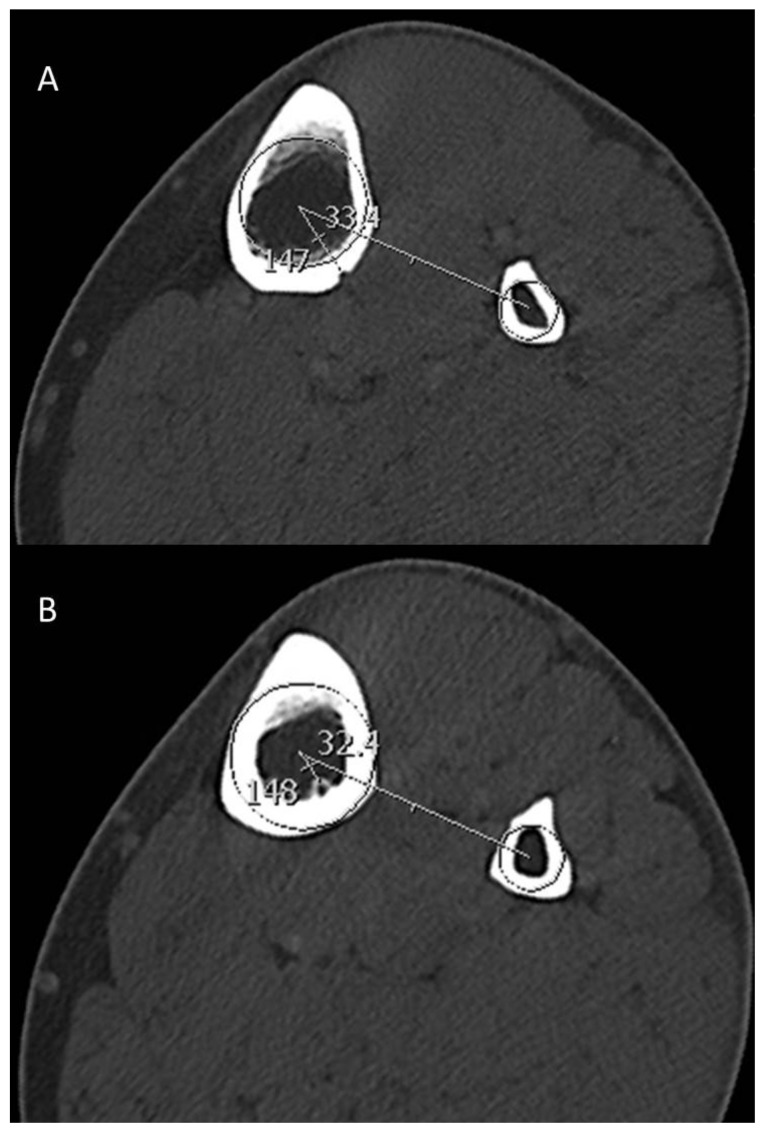
Axial reformatted images in bone window indicating the angular position of the outer foramen of TNAC on the outer cortex (**A**) and the angular position of the inner foramen on the inner cortex (**B**). The angular position is defined by a line connecting the two centers of the tibia and fibula and an intersecting line connecting the center of the tibia to the respective foramen.

-Angular location of outer and inner foramina with respect to the circumference of the tibia using the fibula as the reference point (Figure 3).

-Distance between the outer and inner foramen was defined as length of TNAC.

-TNAC length index: TNAC length relative to total tibial length.

### 2.4. Statistical Analysis

Descriptive statistics were reported as mean ± standard deviation (SD). Unpaired *t*-test was used to compute the differences between men and women. Paired *t*-test was utilized to calculate the differences between the right and left side. The threshold for statistical significance was set at 0.05. SPSS v.25 (Armonk, NY, USA) was utilized for a statistical analysis.

## 3. Results

Between January 2018 and June 2018, a total of 106 consecutive patients (53 males, 53 females), aged 69 ± 12 years (range 42–99) with complete CTAs were included.

Descriptive statistics for the number and location of nutrient foramina for males and females are given in Table 1. Differences between the analyzed anatomical parameters for both genders and sides are given in Table 2.

**Figure 4:**
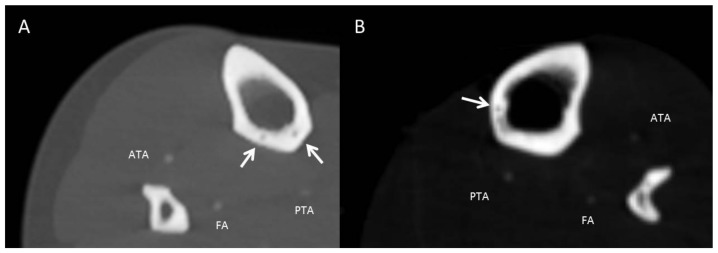
Axial reformatted images in bone window illustrating atypical number and topology of the TNAC. (**A**) Two canals traversing the posterior aspect of the tibia (two white arrows). (**B**) TNAC traversing the medial aspect of the tibia (white arrow). ATA: Anterior tibial artery. PTA: Posterior tibial artery. FA: Fibular artery.

The majority of patients of both genders had only one tibial nutrient canal (~90%), multiple canals or complete absence was rare (Figure 4).

In 96% of cases the outer nutrient foramen was found on the posterolateral aspect of the tibia at about 32% of tibial length measured from tibial plateau for both genders. The inner nutrient foramen was found at about 41–42% of tibial length. The course of nutrient artery canal was mostly cranio-caudal with a small, but significant difference (*p* = 0.012) in relative canal length of 8.5% vs. 10% of tibial length for females and males, respectively. The absolute tibial length was significantly different with 37.4 cm and 36.3 cm for males and females, respectively. The angular location of the inner and outer foramen was between 20–30° and did not reveal a statistically significant difference between genders. No statistically significant differences were found between right and left sides for all analyzed parameters and both genders.

## 4. Discussion

A thorough understanding of the TNAC topography has crucial clinical relevance in the context of trauma surgery, reconstructive surgery, radiographic assessment and bone healing. The tibia is the most commonly fractured long bone in humans and consequently constitutes a huge burden on health-care costs [9,15,16,17,18]. Osseous regions with better vascular supply tend to heal faster than regions with lesser vascularity [15]. In other words, tibial fractures in which the tibial nutrient artery is damaged tend to have an increased incidence of non-union or delayed union [15]. Additionally, iatrogenic damage to the tibial nutrient artery may occur during positioning of internal and external fixation devices hampering fracture healing later on. “Safe corridors” for pin and wire placement have been established, within which iatrogenic injury to nerves and vessels is less likely to occur [19]. However, those classic “safe corridors” of pin placement have been defined to avoid injury to the major neurovascular structures in the lower extremity without considering the TNAC [4,19,20,21]. The position of the pins should classically maintain the stability of the fractured tibia by counteracting the forces which are exerted on the leg (anteroposterior and transverse bending moments) [19,22]. The anteromedial position is usually recommended for pin placement “either in a sagittal plane medial to the tibial crest or perpendicular to the anteromedial surface of the tibia” [21]. The AO Surgery Reference Manual (AO stands for “Arbeitsgemeinschaft für Osteosynthesefragen” and is a world renowned nonprofit organization dedicated to improving the care of people with musculoskeletal injuries) describes a safe corridor with a trajectory angle “(relative to the sagittal plane) of 20–60° for the proximal fragment” [20]. Our study shows that the TNAC mostly traverses in the middle third of the postero-lateral tibia at mean angular location of the inner and outer foramen between 20–30° (Table 2) (Figure 3). Hence, surgeons should exercise caution while inserting pins at the proximal tibia despite the officially recommended “safe corridor”.

In the setting of reconstructive surgery, the use of vascularized allografts has been proven effective albeit challenging [2,23]. Vascularized bone and joint allograft survival is strongly contingent upon preservation of periosteal and intraosseous blood supply [23]. Hence, graft preservation methods as well as surgical techniques depend upon a thorough understanding of vascular anatomy. In this context, Kirchner et al. evaluated 200 tibiae of a central European population regarding the number of tibial nutrient foramina. The authors found one nutrient foramen in 93.5% of the specimens and double foramina in 6.5% [23]. In our study, in 9% of cases a second nutrient canal was identified, but in most cases only a single nutrient canal was present. Double foramina were reported between 0.6–2% in Turkish and South African populations [9,18]. The fact that we found more double foramina than previous studies might be explained by the superiority of CT in comparison to utilizing the naked eye and magnifying lenses in the abovementioned studies (Figure 4). Interestingly, the TNAC was found absent only in one case in our study. The absence of nutrient foramina has been previously observed in an Indian population of 1.4% of studied cases [13]. In this case, the periosteum provides the main vascular blood supply. In light of these findings, the absence of TNAC can be considered rare [10,13].

Most of our findings are in accordance with the findings of Kirchner et al., as well as previous studies conducted in Indian, South African and Turkish populations [3,9,10,18]. The location of the nutrient foramina in the tibia was on the posterior surface at the upper third of the tibia [2,3,9,16,17]. However, there are few reports of anteriorly located nutrient foramina as well [7]. Interestingly, the exact location of the nutrient canal pertaining to the right and left side was similar. Furthermore, the relative circumferential location and cranio-caudal position of the nutrient canal does not show any gender specific differences. The course of the nutrient canal usually runs from a proximal outer foramen to a distal inner foramen which was true for all tibias in our study. This could be explained by the “periostal slipping theory” in which the canal traverses away from the growing end of the long bone [24,25]. In contrast, some studies report a rare caudo-cranial course of the canal which was observed in 0.6% and 1.7% of black and white South Africans, respectively [18]. In our study, the outer foramen was located at the upper-third of the tibial diaphysis which is in accordance with results of a study by Longia et al. [17]; although Campos et al. and Kizilkanat et al. report a position within the middle-third of the tibia [3,9]. The mean tibial length in our study population was well within previously reported ranges of Indian and South African and Turkish populations [9,10,18].

In addition to the TNAC relevance in the context of fracture healing and placement of fixation devices, our results could aid in evaluating longitudinal tibial stress fractures [8,14]. Most longitudinal stress fractures of the tibia (LSFT) nucleate proximally with a transverse or oblique course. Historically, the incidence of LSTF may have been underestimated due to the poor sensitivity of conventional radiographs. The advent of CT and MRI enhanced the detectability of this entity [8,26]. An accurate diagnosis of these fractures is pivotal for safe management. These fractures could be misdiagnosed as an osteomyelitis or as an osseous tumor requiring open biopsy. Consequently, the histological sampling would often reveal osteoid tissue and immature cells mandated by the fracture healing process. This, in turn, would mislead physicians into diagnosing a malignancy. The presence of a lucent fracture line encompassing the osseous cortex and traversing along the diaphyseal axis on several axial images suggests a stress fracture [26,27]. Craig et al. found that LSFT are mostly located on the anteromedial aspect of the tibia [8]. LSTF were described to occur either at the level of the nutrient foramen [8] or superomedial to the foramen [28]. Furthermore, the nutrient foramen may serve a weak point at which longitudinal fractures could nucleate especially if it was atypically located at the anterior aspect of the tibia [8].

### Limitations

The external validity of the results is limited by the sample size, retrospective design and the inherent selection bias. Nevertheless, our sample size is asymptotic to many of the previous studies analyzing the tibial nutrient artery.

Furthermore, this was an analysis on a central European population without ethnic comparisons. A multi-center study with a larger sample size encompassing multiple ethnicities is required to validate the findings of our study. However, our data corroborates the findings published from Indian, South African and Turkish populations. Finally, the quantification of angular position of the nutrient foramina was measured by using the fibula as an internal reference to outmaneuver the variability of patient positioning in the CT scanner. Intraoperatively, patient positioning is standardized [20] and the angle of safe pin positioning is classically measured by using the sagittal plane through the lower leg as a reference.

## 5. Conclusions

This study provides comprehensive data on the morphology and topography of the nutrient foramina and nutrient canal of the tibia. Detailed knowledge of the anatomy could prove useful for clinicians involved in the management of the tibial fractures, vascular graft surgeries as well as radiologic assessment of tibial stress fractures. Most TNAC foramina are located on the posterolateral aspect of the upper third of the tibia with no side specific differences. Significant gender specific differences of the TNAC length may be too small to be of clinical relevance and need to be confirmed in further studies. In addition to neurovascular structures outside the tibial bone, the position of the tibial nutrient artery canal also has to be considered when referring to “safe corridors” of pin/screw insertion for fixation of tibial fractures, in order to safeguard the integrity of tibial blood supply.

## Figures and Tables

**Table 1:** Descriptive statistics for the number of nutrient foramina and location of the inner and outer foramina for both genders.

Parameter	Side		Male (*n* = 53)	Female (*n* = 53)
Number of Foramina	R	0	0 (0)	0 (0)
		1	50 (94)	49 (92.5)
		2	3 (6)	4 (7.5)
		3	0 (0)	0 (0)
	L	0	1 (1.9)	0 (0)
		1	46 (86.8)	51 (96)
		2	5 (9.4)	2 (4)
		3	1 (1.9)	0 (0)
Localization of the Outer Foramen	R	Posterior	52 (98.1)	53 (100)
		Lateral	1 (1.9)	0 (0)
		Medial	0 (0)	0 (0)
	L	Posterior	51 (96.2)	52 (98.1)
		Lateral	1 (1.9)	0 (0)
		Medial	1 (1.9)	1 (1.9)
Localization of the Inner Foramen	R	Posterior	53 (100)	53 (100)
		Lateral	0 (0)	0 (0)
		Medial	0 (0)	0 (0)
	L	Posterior	51 (96.2)	52 (98.1)
		Lateral	1 (1.9)	0 (0)
		Medial	1 (1.9)	1 (1.9)

R = right side; L = left side; values are given as absolute numbers and (percentages).

**Table 2:** Comparison of the different anatomical parameters between males and females as well as the right and left sides.

Parameter	Side	Male	Female	*p* (Male vs. Female)	*p* (R vs. L)
Tibial Length (mm)	R	374 ± 23	363 ± 26	**0.002**	0.59
	L	374 ± 23	364 ± 25		
Distance of Outer Foramen (mm)	R	118 ± 14	113 ± 11	**0.001**	0.69
	L	120 ± 16	113 ± 11		
Distance of Inner Foramen (mm)	R	155 ± 15	144 ± 14	**<0.0001**	0.09
	L	156 ± 16	146 ± 15		
Tibial Nutrient Artery Canal Length (mm)	R	37 ± 12	31 ± 13	**0.004**	0.58
	L	36 ± 12	33 ± 11		
Tibial Nutrient Artery Canal Length Index (%)	R	10 ± 3	8 ± 3	**0.012**	0.32
	L	10 ± 3	9 ± 3		
Outer Foraminal Index (%)	R	32 ± 3	31 ± 3	0.084	0.82
	L	32 ± 4	31 ± 3		
Inner Foraminal Index (%)	R	41 ± 3	40 ± 3	**<0.0001**	0.11
	L	42 ± 4	40 ± 3		
Angular Location of the Outer Foramen (°)	R	26 ± 20	20 ± 13	0.42	0.69
	L	23 ± 16	25 ± 21		
Angular Location of the Inner Foramen (°)	R	29 ± 19	26 ± 11	0.82	0.67
	L	26 ± 14	31 ± 22		

R = right side; L = left side; values are given as arithmetic mean ± standard deviation. Bold denotes statistical significance.
